# Event rates in major Phase 3 heart failure trials over the last 20 years

**DOI:** 10.1093/eschf/xvag169

**Published:** 2026-06-11

**Authors:** Alberto Aimo, Giorgia Panichella, Andrea Ripoli, Guiomar Mendieta Badimon, Faiez Zannad, Michele Emdin

**Affiliations:** Interdisciplinary Center for Health Sciences, Scuola Superiore Sant’Anna, piazza Martiri della Libertà 33, 56127 Pisa, Italy; Cardiology Division, Fondazione Toscana Gabriele Monasterio, via Moruzzi 1, 56124 Pisa, Italy; Cardiology Department, Careggi University Hospital, Largo G.A. Brambilla 3, 50134 Florence, Italy; Cardiology Division, Fondazione Toscana Gabriele Monasterio, via Moruzzi 1, 56124 Pisa, Italy; Department of Cardiology, Hospital de la Santa Creu i Sant Pau, Institut de Recerca Sant Pau (IR SANT PAU), CIBER CV, Universitat Autònoma de Barcelona, Barcelona, Spain; Centro Nacional de Investigaciones Cardiovasculares Carlos III (CNIC), Madrid, Spain; Université de Lorraine, Nancy, France; Interdisciplinary Center for Health Sciences, Scuola Superiore Sant’Anna, piazza Martiri della Libertà 33, 56127 Pisa, Italy; Cardiology Division, Fondazione Toscana Gabriele Monasterio, via Moruzzi 1, 56124 Pisa, Italy

**Keywords:** Heart failure, Therapy, Randomized controlled trials, Mortality, Survival, Trial design

## Abstract

**Introduction:**

To determine whether control-arm event rates in heart failure (HF) randomized clinical trials (RCTs) published in *New England Journal of Medicine* from 2004 to 2024 declined over time, despite intensification of background therapy.

**Methods:**

We identified Phase 3 HF RCTs published in *New England Journal of Medicine* (2004–24). Annualized event rates were calculated as events divided by patients multiplied by follow-up to the primary endpoint. Temporal trends were analysed with weighted least squares (weights equal to the number of control-arm patients), with adjustment for follow-up duration.

**Results:**

Thirty-eight trials met criteria; 31 enrolled HF with reduced ejection fraction (HFrEF; 82%). In HFrEF control arms, median age was 67 years [interquartile range (IQR) 64–69], women were 24% (IQR 21–30), left ventricular ejection fraction (LVEF) was 28% (25–31), N-terminal pro-B-type natriuretic peptide (NT-proBNP) was 1700 ng/L (1273–2879), and the New York Heart Association (NYHA) class III–IV was 46% (29–71). Background therapy included β-blockers 91% (82–93), angiotensin-converting enzyme inhibitors/angiotensin receptor blockers (ARBs)/angiotensin receptor–neprilysin inhibitors 93% (90–97), and mineralocorticoid receptor antagonists (MRAs) 52% (42–59). The median control-arm size was 602 patients (308–1533). Across years, therapy use rose (β-blockers +1.04 percentage points per year, *P* < .001; MRAs +2.05 percentage points per year, *P* < .001), LVEF increased (+.22% per year, *P* = .012), NT-proBNP increased (∼+11.4% per year on the log scale, *P* = .004), and follow-up tended to shorten (*P* = .052). In control arms, all-cause mortality showed no temporal decline [unadjusted slope +.0019 per year; 95% confidence interval (CI) −.0013 to +.0051; *P* = .252]; after adjusting for follow-up, the slope was +.0006 per year (*P* = .711). Longer follow-up was associated with lower annualized mortality (coefficient −.0195 per year; *P* = .032). Cardiovascular mortality was stable (unadjusted +.0003 per year; 95% CI −.0036 to +.0042; *P* = .898; follow-up–adjusted −.0012 per year; *P* = .557). The composite of all-cause death or HF hospitalization increased unadjusted (+.0180 per year; 95% CI +.0063 to +.0298; *P* = .011) but was not significant after follow-up adjustment (+.0113 per year; *P* = .111). Enrichment intensity did not rise linearly (coefficient +.032 criteria per year; *P* = .111), whereas natriuretic-peptide cut-offs were adopted more often [odds ratio (OR) per decade 14.09; 95% CI 1.93–102.75; *P* = .009). Higher age related to higher mortality (coefficient +.0039 per year; *P* = .043). An interaction between year and log-transformed NT-proBNP indicated risk-dependent temporal patterns (*P* = .003).

**Conclusion:**

In major HFrEF trials, control-arm mortality did not decline from 2004 to 2024 despite greater uptake of evidence-based therapy. Risk-enriched enrolment and shorter follow-up likely counterbalanced therapeutic gains.

## Graphical Abstract

**Graphical Abstract:**
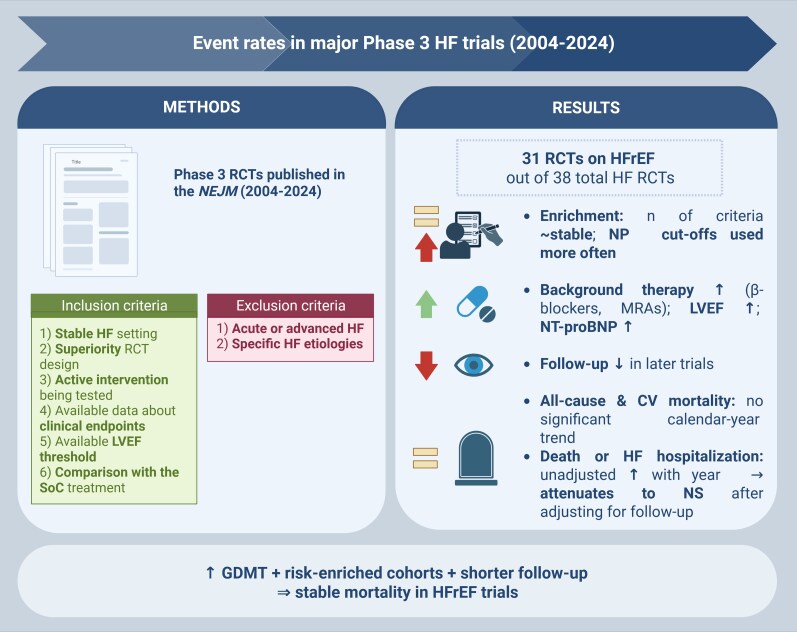
Mortality in heart failure randomized clinical trials. See text for details. CV, cardiovascular; GDMT, guideline-directed medical therapy; HFrEF, HF with reduced ejection fraction; LVEF, left ventricular ejection fraction; NEJM, *New England Journal of Medicine*; NP, natriuretic peptide; NS, non-significant; SoC, standard-of-care.

## Introduction

Over the last decades, the therapeutic landscape of heart failure (HF) has undergone dramatic changes, which have translated into improved prognosis for many patients with HF. Landmark randomized clinical trials (RCTs) conducted in the late 1990s and early 2000s demonstrated that β-blockers, angiotensin-converting enzyme inhibitors (ACEi), and angiotensin receptor blockers (ARBs) significantly reduced mortality in HF with reduced ejection fraction (HFrEF), prompting their widespread adoption into clinical practice.^1–6^ Subsequent trials demonstrated the efficacy of additional strategies, such as mineralocorticoid receptor antagonists (MRAs),^7^ device therapies [e.g. implantable cardioverter defibrillator (ICD) and cardiac resynchronization therapy (CRT)],^8–10^ and more recently, angiotensin receptor–neprilysin inhibitors (ARNIs)^11^ and sodium–glucose cotransporter-2 inhibitors (SGLT2i).^12,13^ Heart failure with mildly reduced or preserved ejection fraction (HFmrEF/HFpEF) have received increasing consideration over the years. Neurohormonal drugs have been recommended even in HFmrEF and even HFpEF, despite the lack of strong evidence,^14,15^ and are increasingly prescribed. These advances in HF therapy over time have been accompanied by a consistent decrease in mortality among patients with HF across age groups.^16,17^

From a trial design perspective, improvements in HF outcomes create unique challenges. As all-cause mortality declines, trials require larger patient cohorts and/or longer follow-up duration to detect statistically significant differences in mortality benefit between treatment arms. In parallel, trialists have increasingly employed enrichment strategies to ensure that events (particularly death or hospitalization) occur at a sufficient rate to power their studies. By strategically recruiting higher-risk populations, event rates in such trials may be maintained or even increased, counteracting the overall trend of declining mortality due to improved background therapy.

Although prior analyses have demonstrated a decrease in sudden cardiac death throughout the 2000s,^18^ a broader evaluation of how overall event rates have changed in major Phase 3 HF trials over the last 20 years is lacking. In this analysis, we reviewed and quantified the rates of mortality in major Phase 3 HF RCTs published from 2004 to 2024. We highlight the characteristics of the study populations, the background use of evidence-based therapies, and the enrichment strategies used. We aimed to determine whether event rates in HF trials have declined over the past two decades, or whether these rates have been preserved by the increasing reliance on enrolment criteria that select the higher-risk patients. Such insights are important for refining the design of future HF clinical trials and ensuring they remain adequately powered to detect meaningful clinical benefits.

## Methods

### Eligibility criteria

Phase 3 RCTs published in an issue of the *New England Journal of Medicine* (*NEJM*) from 1 January 2004 to 31 December 2024 were included if they met the following criteria: (i) HF setting, (ii) stable HF patients, excluding those with acute or advanced HF, as defined by the trialists, (iii) superiority RCT design, (iv) trials testing a drug, device, interventional procedure, or surgery, (v) providing data about clinical endpoints (at least all-cause mortality), (vi) defining an EF threshold for patient inclusion, and (vii) with a follow-up duration of at least 1 year. Additionally, RCTs had to involve a comparison with the standard of care treatment at the time of the trial or comparison with a drug or device that has since become part of the standard of care. For example, a trial comparing an intervention with defibrillator therapy for patients with EF ≤35% could be included, while a trial comparing ICD therapy with amiodarone could not. Finally, trials on patients with HF and a specific comorbidity (e.g. obesity or atrial fibrillation) were included, whereas trials on specific HF aetiologies (e.g. amyloid cardiomyopathy) were not.

Two authors (A.A. and G.P.) independently screened all the original articles from *NEJM* issues available in the online archive (https://www.nejm.org/loi/nejm). For articles on the topic of HF, the full text was screened. Any disagreements in article selection were resolved by evaluation of a third author (M.E.).

### Data extraction: study design and enrichment criteria

Data extraction was undertaken independently by two authors (A.A. and G.P.) using a standardized data collection form. The following data related to study design were retrieved: EF range, additional criteria to define the study setting (e.g. QRS ≥120 ms and PR >150 ms),^1^ age range, the New York Heart Association (NYHA) class, N-terminal pro-B-type natriuretic peptide (NT-proBNP) cut-off for inclusion, prior recent HF hospitalization and/or urgent HF visit, need of current diuretic therapy, and additional inclusion criteria. The following inclusion criteria were considered as ‘enrichment criteria’ (i.e. patient characteristics identifying individuals with a higher likelihood of events): NYHA class III–IV, any age threshold ≥40 years, any natriuretic peptide cut-off, prior recent HF events, and pulmonary congestion or elevated filling pressures. The number of enrichment criteria used in each trial was reported. When two criteria could be present alone or in combination (e.g. ‘elevated natriuretic peptide’ OR ‘recent HF event’), only one criterion was counted.

### Data extraction: population characteristics and endpoints

The following data were then extracted: number of patients in each study arm, mean/median age, EF, NT-proBNP, and percentage of female patients and of patients in NYHA class III or IV. Patients were also classified according to the current treatment paradigm for HF care^2^ as those receiving β-blockers, ACEi/ARB/ARNI, MRA, SGLT2i, ICD, or CRT.

For each study, data about primary endpoint and mean/median follow-up duration to that endpoint were retrieved, as well as the number of patients experiencing all-cause mortality, cardiovascular mortality, or additional endpoints (all-cause mortality or first HF hospitalization; cardiovascular mortality or first HF hospitalization; first HF hospitalization).

### Statistical analysis

Statistical analysis was performed using IBM SPSS Statistics (version 22, 2013), R (version 4.2.2), and Python (version 3.x; pandas, numpy, statsmodels, matplotlib). Normal distribution was assessed through the Shapiro–Wilk test; as all variables were non-normally distributed, they were presented as median and interquartile interval. Missing arm-level data were not imputed; models used complete cases. Between-group comparisons of descriptive characteristics (e.g. HFrEF vs HFmrEF/HFpEF) used Welch’s *t*-test or Mann–Whitney for continuous variables and Fisher’s exact or χ^2^ (with Yates correction, when applicable) for categorical variables. Annualized rates were computed as events ÷ (number of patients × follow-up duration in years). For mortality, the follow-up used was the trial-reported time to the primary endpoint (time-to-first event) rather than time to all-cause death, not provided in the original studies. To evaluate temporal trends in control-arm event rates, we regressed annualized rates on calendar year of study start using weighted least squares (WLS) with weights equal to the control-arm sample size (*N*control), and WLS additionally adjusting for follow-up duration. Slopes (β per calendar year), 95% confidence intervals (CIs), and *P* values are reported. Parallel models were fit for cardiovascular mortality and the composite of all-cause death or HF hospitalization. Sensitivity models simultaneously included study size and follow-up. For HFrEF trials, baseline cohort features (age, % women, LVEF, log-transformed NT-proBNP, % NYHA III–IV, and background therapy: % on β-blocker, ACEi/ARB/ARNI, and MRA) were regressed on calendar year using WLS (weights = *N*control); slopes reflect change per year. NT-proBNP was analysed on the log scale. The number of enrichment criteria (NYHA III–IV; any age threshold ≥40 years; any natriuretic peptide cut-off; prior recent HF event; pulmonary congestion/elevated filling pressures) was modelled vs year with WLS. For each individual criterion (present/absent), we ran logistic models with year as a continuous predictor, reporting odds ratios per decade and, complementarily, linear-probability estimates as percentage-point change per year. We also performed an era comparison using a pre-specified cut-point at 2008 (median year of study start in the dataset), reporting criterion adoption as counts/percentages, odds ratios with 95% CIs, risk differences (late–early, percentage points), and Fisher’s exact *P* values. For the number of criteria, eras were compared using Welch’s *t*-test (mean difference with 95% CI) and Mann–Whitney tests for medians. To examine how baseline characteristics and enrichment relate to event rates (and to the year effect), we used WLS add-one models of the form: endpoint ∼ year + follow-up + predictor (weights = *N*control). For each predictor, we report its coefficient and *P-*value, Δ*R*^2^ vs the base model (year + follow-up), and the change in the year coefficient (attenuation/amplification). Predictors with *P* < .10 in add-one screening were entered (capped to limit overfitting) into a compact multivariable WLS model per endpoint. Effect modification was assessed with centred interaction models: endpoint ∼ year_c + follow-up + predictor_c + (year_c×predictor_c), where year_c and predictor_c are mean-centred; we report the interaction term and the year slope at the mean predictor. Given limited arm-level sample sizes, interactions were flagged at *P* < .10; all other tests used two-tailed *P* < .05. No multiplicity adjustments were applied.

## Results

### Heart failure with reduced ejection fraction vs heart failure with reduced ejection fraction trials

From an estimated 4368 original articles (4 original articles per issue, 52 issues per year, 21 years) we identified 75 original articles on the topic of HF. After exclusion of 36 articles (Supplementary Figure S1), we ultimately selected 38 RCTs, ^9–13,19–50^ whose main design features are reported in *Table 1*. The majority of RCTs (*n* = 31, 82%) were on HFrEF (as defined by the trialists), and the other 7 (18%) on HFmrEF/HFpEF. In RCTs on HFrEF, the upper reference limit of LVEF was more often 35% (*n* = 13, 42%), followed by LVEF 40% (*n* = 9, 29%). Trials on HFmrEF/HFpEF had more often a lower LVEF threshold of 45% (*n* = 4, 57%), the others adopting the threshold of 41% (*n* = 2, 29%), or 40% (*n* = 1, 14%). Heart failure with mildly reduced or preserved ejection fraction trials were started more recently and were larger than HFrEF trials, and most baseline characteristics differed; conversely, follow-up duration did not differ significantly (Supplementary Table S1). Given their predominance and the substantial heterogeneity of HFmrEF/HFpEF RCTs, the following analyses were conducted just on HFrEF trials.

**Table 1:** Randomized controlled trials in heart failure: main study design features

RCT [ref.]	Year study initiation	Comparison	LVEF range	Additional criteria to define study setting	Age range (years)	NYHA	NT-proBNP cut-offs	Previous HF event	Diuretic therapy	Additional criteria	Primary endpoint
COMPANION^19^	2000	CRT vs standard of care	≤35%	QRS ≥120 ms and PR >150 ms		III–IV		HF hospitalization ≤12 months			All-cause mortality or all-cause hospitalization
DEFINITE^20^	1998	ICD vs standard of care	≤35%	Non-ischaemic DCM, ventricular arrhythmias		<IV					All-cause mortality
A-HeFT^21^	2001	Isosorbide dinitrate plus hydralazine vs placebo	≤35% or ≤45% plus LV dilation	Black	≥18	III–IV					Composite score (weighted values for all-cause mortality, HF hospitalization, change in the quality of life)
SCD-HeFT^9^	1997	ICD vs amiodarone vs placebo	≤35%		≥18	II–III					All-cause mortality
CARE-HF^10^	2001	CRT vs standard of care	≤35%	Cardiac dyssyncrhony	≥18	III–IV				LV end-diastolic dimension ≥30 mm (indexed to height), QRS ≥120 ms	All-cause mortality or CV hospitalization
CANPAP^22^	1998	CPAP vs standard of care	<40%	Central sleep apnoea	18–79	II–IV					All-cause mortality or heart transplantation
CORONA^23^	2003	Rosuvastatin vs placebo	≤40% (≤35% if NYHA II)		≥60	II–IV				Ischaemic HF	CV mortality, nonfatal MI, nonfatal stroke
Atrial Fibrillation and Congestive Heart Failure^24^	2001	Rhythm vs rate control	≤35%	History of ‘congestive HF’, history of AF	≥18			HF hospitalization ≤6 months		Pulmonary congestion on radiography, LV hypertrophy or left atrial enlargement on echo, or LV hypertrophy or LBBB on ECG	All-cause mortality or CV hospitalization
I-PRESERVE^25^	2002	Irbesartan vs placebo	≥45%		≥60	II–IV		HF hospitalization ≤6 months		Pulmonary congestion on radiography, LV hypertrophy or left atrial enlargement, LV hypertrophy or LBBB on ECG	All-cause mortality or CV hospitalization
MADIT-CRT^26^	2004	CRT-D vs ICD (current standard of care)	≤30%	QRS ≥130 ms	≥21	I-II (ischaemic), II (non-ischaemic)					All-cause mortality, nonfatal HF event
FAIR-HF^27^	2007	Ferric carboxymaltose vs placebo	≤40% (if NYHA II) or 45% (if NYHA III)	Hb 9.5–13.5 g/dL, iron deficiency		II–III					Self-reported Patient Global Assessment and NYHA functional class at week 24
RAFT^28^	2003	CRT vs standard of care	≤30%	QRS ≥120 ms or paced QRS ≥200 ms		II–III (only II after amendment)					All-cause mortality or HF hospitalization
EMPHASIS-HF	2006	Eplerenone vs placebo	≤35% (with QRS >130 ms if 31–35%)		≥55	II	BNP ≥250 ng/L or NT-proBNP ≥500 ng/L (M) or 750 ng/L (W) if no CV hospitalization <6 months				CV mortality or HF hospitalization
STICH^29^	2002	CABG vs standard of care	≤35%	CAD suitable for CABG	≥18						All-cause mortality
RED-HF^30^	2006	Darbepoetin α vs placebo	≤40%	Hb 9–12 g/dL		II–IV					All-cause mortality or HF hospitalization
BLOCK HF^31^	2003	Biventricular pacing vs right ventricular pacing (current standard of care)	≤50%	High-degree AV block		I-III					All-cause mortality, urgent HF visit, ≥15% increase in LVESVi
EchoCRT^32^	2008	CRT-D vs ICD (current standard of care)	≤35%	QRS <130 ms + LV dyssynchrony	≥18	III–IV					All-cause mortality or HF hospitalization
TOPCAT^33^	2006	Spironolactone vs placebo	≤45%		≥50	(≥1 HF symptom)	‘Unexplained’ BNP ≥100 ng/L or NT-proBNP ≥360 ng/L	≥1 HF hospitalization ≤12 months			CV mortality, aborted cardiac arrest, HF hospitalization
PARADIGM-HF^11^	2009	Sacubitril/valsartan vs enalapril	≤40% (≤35% after amendment)		≥18	II–IV	BNP ≥150 ng/L or NT-proBNP ≥600 ng/L or (if HF hospitalization <12 months) BNP ≥100 ng/L or NT-proBNP ≥400 ng/L				CV mortality, HF hospitalization
SERVE-HF^34^	2008	ASV vs standard of care	≤45%		≥22	III–IV, or II with ≥1 hospitalization for HF in the last 24 months					All-cause mortality, cardiac transplantation, VAD implantation, resuscitation after sudden cardiac arrest, or appropriate lifesaving shock, HF hospitalization
ATMOSPHERE^35^	2009	Aliskiren vs enalapril vs aliskiren vs aliskiren + enalapril	≤35%		≥18	II–IV	BNP ≥150 ng/L (or BNP ≥100 ng/L + unplanned HF hospitalization ≤12 months) or NT-proBNP ≥600 ng/L (or NT-proBNP ≥400 ng/L + unplanned HF hospitalization ≤12 months)				CV mortality, HF hospitalization
DANISH^36^	2008	ICD vs standard of care	≤35%	Non-ischaemic HF		II–III (or IV if CRT planned)	NT-proBNP >200 ng/L				All-cause mortality
COMMANDER HF^37^	2013	Rivaroxaban vs placebo	≤40%	Significant CAD, no AF	≥18		BNP ≥200 ng/L or NT-proBNP ≥800 ng/L	Previous HF decompensation	Required		All-cause mortality, MI, stroke
CASTLE-HF^38^	2008	Catheter ablation vs medical therapy	≤35%	Symptomatic paroxysmal or persistent AF	≥18	II–IV					All-cause mortality or HF hospitalization
MITRA-FR^39^	2013	Percutaneous repair of mitral regurgitation or medical therapy	15–40%	Secondary MR	>18	II–IV		HF hospitalization ≤12 months			All-cause mortality or HF hospitalization at 12 months
COAPT^40^	2012	Percutaneous repair of mitral regurgitation or medical therapy	20–50%	Secondary MR	≥18	II–IV	BNP ≥300 ng/L or NT-proBNP ≥1500 ng/L	HF hospitalization ≤12 months			HF hospitalization at 24 months
PARAGON-HF^41^	2014	Sacubitril/valsartan vs valsartan	≥45%		≥50	II–IV	NT-proBNP >300 ng/L without AF or >900 ng/L with AF	HF hospitalization ≤9 months	≥30 days	Structural heart disease	CV mortality, total HF hospitalization
DAPA-HF^12^	2017	Dapagliflozin vs placebo	≤40%		≥18	II–IV	NT-proBNP ≥600 ng/L or ≥400 ng/L if HF hospitalization <12 months; ≥900 ng/L if AF				CV mortality, worsening HF
VICTORIA^42^	2016	Vericiguat vs placebo	<45%		≥18	II–IV	SR: NT-proBNP ≥1000 ng/L, BNP ≥300 ng/L; AF: ≥1600 ng/L, ≥500 ng/L	HF hospitalization ≤6 months or IV diuretic ≤3 months			CV mortality, HF hospitalization
EMPEROR-Reduced^13^	2017	Empagliflozin vs placebo	≤40%		≥18	II–IV	If EF ≥36% to ≤40%: NT-proBNP ≥2500 ng/L without AF, ≥5000 ng/L with AF b) If EF ≥31% to ≤35%: NT-proBNP ≥1000 ng/L without AF, OR ≥2000 ng/L with AF c) If EF ≤ 30%: NT-proBNP ≥600 ng/L without AF, ≥1200 ng/L with AF d) For EF ≤ 40% and HF hospitalization ≤12 months, NT-proBNP ≥600 ng/L without AF, ≥1200 ng/L with AF				CV mortality, HF hospitalization
EMPEROR-Preserved^43^	2017	Empagliflozin vs placebo	>40%		≥18	II–IV	NT-proBNP ≥300 ng/L or 900 ng/L if AF	HF hospitalization ≤12 months	(not mandatory)	Structural heart disease	CV mortality, HF hospitalization
DELIVER^44^	2018	Dapagliflozin vs placebo	>40%		≥40	II–IV	NT-proBNP ≥300 ng/L in SR or ≥600 ng/L if AF/flutter		At least intermittent diuretic therapy	Structural heart disease	CV mortality, worsening HF
REVIVED-BCS^45^	2013	Percutaneous revascularization vs OMT	<35%		≥18						All-cause mortality, HF hospitalization
HEART-FID^46^	2017	iv ferric carboxymaltose or placebo	≤40%	Iron deficiency	≥18	II–IV	NT-proBNP >600 ng/L or >1000 ng/L with AF	HF hospitalization ≤12 months			All-cause mortality, HF hospitalization or change in 6MWD
CASTLE HTx^47^	2020	Catheter ablation vs medical therapy	≤35%	Symptomatic AF, end-stage HF referred for transplantation evaluation	≥18	II–IV					All-cause mortality, VAD implantation, urgent heart transplantation
STEP HFpEF^48^	2021	Semaglutide vs placebo	≥45%	BMI ≥30	≥18	II–IV	≥220 ng/L (for patients with BMI <35.0 and SR), ≥660 ng/L (for patients with BMI <35.0 and AF), ≥125 ng/L (for patients with BMI ≥35.0 and SR), or ≥375 ng/L (for patients with BMI ≥35.0 and AF)	HF hospitalization ≤12 months		Elevated LV filling pressure	Change from baseline in the KCCQ-CSS; change in body weight
FINEHEARTS^49^	2020	Finerenone vs placebo	≥40%		≥40	II–IV	NT-proBNP ≥300 ng/L (BNP ≥100 ng/L) in SR or NT-proBNP ≥900 ng/L (BNP ≥300 ng/L) in AF		≥30 days	Structural heart disease	CV mortality, worsening HF
RESHAPE-HF2^50^	2015	Percutaneous repair of mitral regurgitation or medical therapy	20–50%	Moderate to severe functional MR	18–90	II–IV	BNP ≥300 ng/L or NT-proBNP ≥1000 ng/L	HF hospitalization ≤12 months + HF hospitalization ≤90 days before enrolment			CV mortality and HF hospitalization for HF at 24 months; HF hospitalization at 24 months; change from baseline in the KCCQ-OS score at 12 months

Trials were ordered based on their publication date. 6MWD, 6-min walking distance; AF, atrial fibrillation; AV, atrio-ventricular; BMI, body mass index; BNP, B-type natriuretic peptide; CAD, coronary artery disease; CPAP, continuous positive airway pressure; CRT, cardiac resynchronization therapy; CV, cardiovascular; DCM, dilated cardiomyopathy; ICD, implantable cardioverter defibrillator; i.v., intravenous; KCCQ, Kansas City Cardiomyopathy Questionnaire (CSS, Clinical Summary Score; OS, Overall Summary), LBBB, left bundle branch block; LV, left ventricle; LVEF, left ventricular ejection fraction; MI, myocardial infarction; MR, mitral regurgitation; NT-proBNP, N-terminal pro-B-type natriuretic peptide; NYHA, New York Heart Association; OMT, optimal medical therapy; SR, sinus rhythm; VAD, ventricular assist device.

### Event rates across calendar years in heart failure with reduced ejection fraction trials

**Figure 1:**
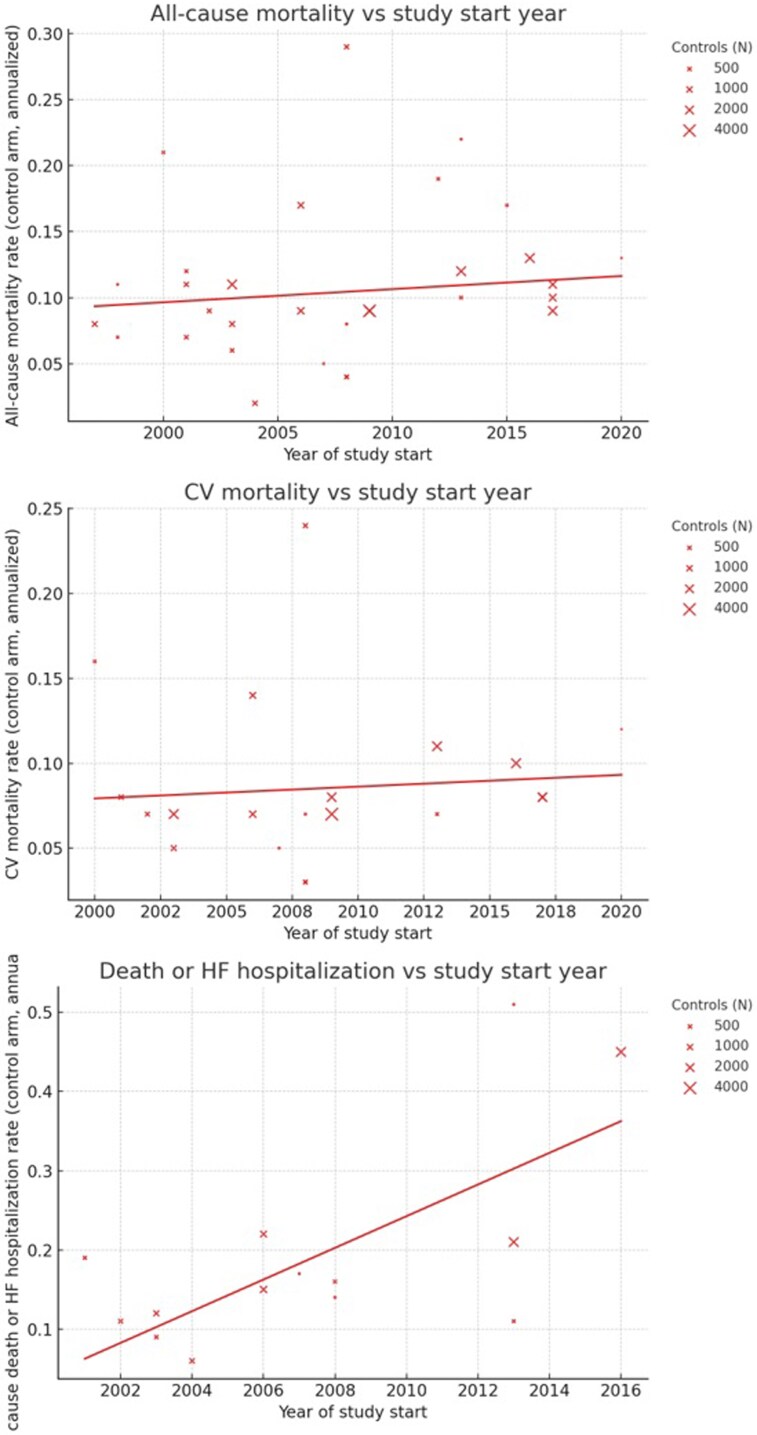
Weighted least squares regressions of annualized control-arm event rates vs year of study start. (Above) All-cause mortality, (middle) cardiovascular mortality, and (below) all-cause death or heart failure hospitalization. Each cross represents one trial; marker size is proportional to the number of patients in the control arm (see size legend). The solid line is the weighted least squares fit (weights = control-arm sample size). Event rates are annualized as events ÷ (patients × follow-up to the primary endpoint).

In a simple linear regression of annualized all-cause death rates in the control arms on year of study start, the slope was small and not significant (β = +.0019 per year; 95% CI −.0013 to +.0051; *P* = .252). Accounting for study size yielded similar, non-significant estimates (β = +.0010, *P* = .453) (*Figure 1*). Follow-up duration tended to decrease from the earliest to the latest trials (*P* = .052, β = −.352). Adjusting for follow-up duration further attenuated the year effect (β = +.0006, *P* = .711), while follow-up duration itself was inversely associated with annualized mortality (β = −.0195 per additional year; *P* = .032). In a model including both study size and follow-up, the year effect remained non-significant (β = +.0010, *P* = .545).

In linear regression of annualized cardiovascular death rates from control arms on the year of study start (*n* = 20), the slope was small and non-significant (β = +.0003 per year; 95% CI −.0036 to +.0042; *P* = .898). Adjusting for study size yielded similarly non-significant estimates (β = +.0007, *P* = .659). After adjusting for follow-up duration, the year effect remained non-significant (β = −.0012, *P* = .557), while follow-up duration was inversely associated with annualized cardiovascular mortality (β = −.0180 per additional year; *P* = .049), with similar results when also adjusting for study size (β = −.0183; *P* = .053).

In linear regression of annualized rates of all-cause mortality or HF hospitalization from control arms on year of study start (*n* = 14), the unadjusted slope was positive and significant (β = +.0180 per year; 95% CI +.0063 to +.0298; *P* = .011), with similar results when using size-weighted models (β = +.0200, *P* = .001). After accounting for follow-up duration, the calendar-year effect attenuated and was no longer significant (β = +.0113, *P* = .1107), while follow-up duration itself showed an inverse association with the annualized composite rate (β = −.0452 per additional year; *P* = .084), with comparable findings in the model including both size and follow-up (β = −.0452, *P* = .101).

### Baseline characteristics across calendar years

We then investigated the changes in study cohorts across the years of study start (*Table 2*). Calendar year was associated with a clear intensification of background therapy and shifts in clinical profile. β-Blocker use increased by 1.04 percentage points (pp) per year (β = 1.04; 95% CI .74–1.33; *P* < .001), and MRA use by 2.05 pp/year (β = 2.05; 95% CI 1.42–2.69; *P* < .001), whereas ACEi/ARB/ARNI showed no clear temporal change (β = −.26 pp/year; 95% CI −.59 to .07; *P* = .134). Baseline LVEF rose modestly over time (+.22 EF%/year; 95% CI .06–.38; *P* = .012). NT-proBNP increased; on the log scale this corresponds to +12.1% per year (95% + 5.0% to +19.7%; *P* = .004). Demographic and symptom-class measures were stable: age (β = +.09 years/year; *P* = .456), women (β = +.06 pp/year; *P* = .768), and NYHA III–IV (β = −.62 pp/year; *P* = .418) showed no significant trends.

**Table 2:** Baseline characteristics vs year of study start

Variable (unit)	Raw/log-transformed	No. of trials	β per year	CI low	CI high	*P*-value
**Age (years)**	Raw	31	.0879	−.1400	.3158	.456
**Women (percentage points)**	Raw	31	.0627	−.3497	.4750	.768
**LVEF (percentage points)**	Raw	31	.2206	.0585	.3828	.012
**NT-proBNP (ng/L)**	Raw	16	98.8823	12.7202	185.0444	.041
**NT-proBNP (percentage change)**	Log	16	.1142	.0490	.1794	.004
**NYHA III–IV (percentage points)**	Raw	29	−.6230	−2.1067	.8606	.418
**β-Blocker use (percentage points)**	Raw	30	1.0371	.7408	1.3334	0
**ACEi/ARB/ARNI use (percentage points)**	Raw	30	−.2616	−.5935	.0702	.134
**MRA use (percentage points)**	Raw	26	2.0547	1.4243	2.6852	0

Weighted least squares regression analysis, weighing for the number of patients in the control arm. For continuous variables, mean or median values in the control arm were computed. ACEi/ARB/ARNI, angiotensin-converting enzyme inhibitor/angiotensin receptor blocker/angiotensin receptor/neprilysin inhibitor; FU, follow-up; LVEF, left ventricular ejection fraction; MRA, mineralocorticoid receptor antagonist; NT-proBNP, N-terminal pro-B-type natriuretic peptide; NYHA, New York Heart Association.

### Enrichment criteria across calendar years

The number of enrichment criteria did not increase significantly over calendar time (β = +.032 criteria per year; 95% CI −.008 to +.073; *P* = .111; Supplementary Table S2). Logistic models assessing individual criteria showed time-related increases in the use of natriuretic-peptide cut-offs and other risk-enrichment requirements (Supplementary Table S2): odds ratios per decade were >1 for most criteria, with linear probability estimates indicating positive percentage-point changes per year. In era comparisons split at 2008, adoption rates for enrichment features were uniformly higher in the later era, consistent with broader use of peptide thresholds and clinical-risk requirements in contemporary trial designs (Supplementary Table S3).

### Baseline characteristics, enrichment criteria, and event rates across calendar years

In weighted models adjusting for follow-up, several cohort/design features related to control-arm event rates. For all-cause mortality, age was positively associated with annualized rates (β = .004 per year of age; *P* = .043; Δ*R*^2^ = .110), with a modest attenuation of the year slope (−7.9% vs the base model). For cardiovascular mortality, no single predictor reached *P* < .05; however, trials requiring NYHA III–IV at entry showed a clear trend towards higher CV death rates (β = +.064; *P* = .073; Δ*R*^2^ = .143). For the composite of all-cause death or HF hospitalization, ACEi/ARB/ARNI use in control arms was inversely associated with event rates (β = −.017 per percentage-point; *P* = .032; Δ*R*^2^ = .115) and attenuated the positive year slope by 22% (Supplementary Table S4).

Forest plots of the calendar-year slopes under sequential model, specifications are shown in Supplementary Figure S2. All-cause and cardiovascular mortality slopes clustered around zero across models, whereas the unadjusted increase over time for the composite endpoint attenuated after accounting for follow-up and covariates.

Effect-modification analyses further indicated that the apparent calendar-year signal depends on baseline risk and treatment mix: for all-cause mortality, a strong year*log(NT-proBNP) interaction (*P* = .003; Δ*R*^2^ = .45) reduced the year coefficient from −.00158 (base) to −.00008 at mean NT-proBNP, suggesting that differences in baseline risk capture much of the temporal variation. For the composite endpoint, year*percentage of women (β-interaction = +.00037; *P* = .002) and year*LVEF (β-interaction = −.00031; *P* = .010) were significant, with additional weaker interactions for percentage of patients with NYHA class III–IV (*P* = .058), ACEi/ARB/ARNI use (*P* = .071), and the number of enrichment criteria (*P* = .078) (Supplementary Table S5).

## Discussion

In this analysis of 38 major Phase 3 HF trials published between 2004 and 2024, most of which enrolled patients with HFrEF, we found that control-arm all-cause mortality has not declined over time, despite the secular uptake of evidence-based therapies and the availability of newer agents. In simple regressions, the calendar-year slope for all-cause death was small and non-significant; weighting by study size yielded similar estimates, and additional adjustment for follow-up further attenuated the year effect. Cardiovascular mortality showed the same pattern. By contrast, the unadjusted composite of all-cause death or HF hospitalization increased over time but lost significance after accounting for follow-up, underscoring the central role of exposure time when interpreting annualized rates. Overall, these observations point to no convincing secular decline in mortality within trial control arms once design features are considered.

A likely explanation lies in countervailing forces that have reshaped trial populations. On the one hand, background therapy intensified: β-blocker and MRA use rose markedly across calendar years, consistent with contemporary care; on the other, several features indicate risk enrichment. Baseline LVEF and NT-proBNP increased over time (the latter on the log scale), and the use of natriuretic peptide thresholds as inclusion criteria became more common, even as the total number of enrichment criteria did not significantly rise. Importantly, a greater number of enrichment criteria predicted higher all-cause mortality, confirming that eligibility strategies successfully concentrate risk. These dynamics (more treatment but also more risk-selected cohorts) help explain why mortality rates remained stable rather than declining.

Follow-up duration tended to shorten over the two decades. Because our rates are annualized as events ÷ (patients × years) using time to the primary endpoint (often a composite) when mortality-specific follow-up was unavailable, recent trials with shorter follow-up are prone to undercount late deaths. The assumption of roughly constant hazards inherent to annualization may therefore underestimate mortality in newer studies. In this context, the observed stability of all-cause mortality should be interpreted as conservative: true mortality is certainly not decreasing and could be flat or higher after harmonizing follow-up windows.

Our interplay analyses reinforce this interpretation. In add-one WLS models adjusting for follow-up, older age and higher NYHA III–IV burden tracked with higher all-cause mortality, whereas ACEi/ARB/ARNI use tracked with lower composite event rates; requiring NYHA III–IV as a formal enrichment criterion was associated with higher CV death. Moreover, interaction models showed that the apparent calendar-year signal depends on baseline risk and therapy mix: for all-cause mortality, a year × log(NT-proBNP) interaction substantially reduced the year coefficient at the mean NT-proBNP, and for the composite, significant year*women% and year*LVEF interactions indicate that temporal trends differ across demographic and physiologic strata. Together, these findings suggest that secular differences in risk profiles and treatment penetration, rather than calendar time *per se*, largely account for the stability of mortality and the attenuation of the composite trend.

These trends have design and interpretability consequences. First, when follow-up is abbreviated, composite endpoints dominated by early hospitalizations may appear more ‘dynamic’ over time than mortality, which accrues later; powering assumptions should reflect this timing. Second, eligibility strategies (e.g. NP cut-offs, recent HF events, NYHA III–IV requirements) efficiently raise event rates but can shift representation away from subgroups with different biology and risk horizons. Fixed natriuretic peptide thresholds, for instance, may under-enrol women, older adults, patients with high body mass index (BMI), and HFpEF phenotypes who often have lower peptide levels for a given risk; conversely, requirements for recent hospitalization may under-represent chronically symptomatic outpatients or institutionalized older adults managed outside hospitals. Sponsors and investigators might therefore consider context-adjusted NP thresholds (e.g. by rhythm or BMI), stratified caps/targets for age and sex, deliberate oversampling of HFpEF/HFmrEF strata, and pre-specified subgroup power to ensure that inferences extend to populations commonly seen in practice.

Several limitations must be acknowledged. We focused on RCTs published in *NEJM* to maximize reporting consistency and methodological quality. While this enhances comparability, it may introduce selection bias towards larger, practice-changing, or more intensively adjudicated trials with high background therapy uptake. Inclusion of Phase 3 HF trials from other leading journals (e.g. *The Lancet*, *JAMA*, *European Heart Journal*, *Circulation*) would broaden geography, care settings, and intervention mix, potentially shifting observed event rates and altering apparent temporal stability. Additionally, our arm-level approach required using primary endpoint follow-up as the exposure time for mortality when death-specific follow-up was unavailable, and we applied no multiplicity adjustment to exploratory modelling (predictor screening and interactions), which should be viewed as hypothesis generating. Access to individual-patient data would enable more granular adjustment (e.g. time-to-event modelling with patient-level covariates) and improve precision around effect modification.

## Conclusions

Across two decades of major HFrEF trials, control-arm mortality did not decline, a finding best explained by the offsetting effects of intensifying background therapy and risk-enriched enrolment, compounded by shorter follow-up in recent trials that likely underestimates mortality. Future trial designs should balance the need for events with external validity, adopt follow-up durations appropriate to capture late mortality, and incorporate prospective strategies (stratified enrolment, context-adjusted thresholds, and powered subgroup analyses) to ensure that results are generalizable to older adults, women, and HFpEF populations.

## Supplementary Material

xvag169_Supplementary_Data

## Declarations

### Data Availability

No data were generated or analysed for this manuscript.

### Pre-registered Clinical Trial Number

None supplied.
