# Supplementary material for: Event rates in major Phase 3 heart failure trials over the last 20 years
Source: ESC Heart Fail. 2026 Jun 11;13(3):xvag169. doi: 10.1093/eschf/xvag169 (PMC13298876; doi:10.1093/eschf/xvag169)
Supplement: xvag169_Supplementary_Data [file xvag169_supplementary_data.docx]

**Supplementary material**

**Event rates in major phase 3 heart failure trials over the last 20 years**

Alberto Aimo^1,2^, Giorgia Panichella^3^, Andrea Ripoli^2^, Guiomar Mendieta Badimon^4^, Faiez Zannad^5^, Michele Emdin^1,2^

1. Interdisciplinary Center for Health Sciences, Scuola Superiore Sant’Anna, Pisa, Italy; 2. Cardiology Division, Fondazione Toscana Gabriele Monasterio, Pisa, Italy; 3. Careggi University Hospital, Florence, Italy; 4. Hospital Clínic de Barcelona, Barcelona, Spain; 5. Université de Lorraine, Nancy, France.

**Supplemental Figure 1. Flowchart of study selection.**


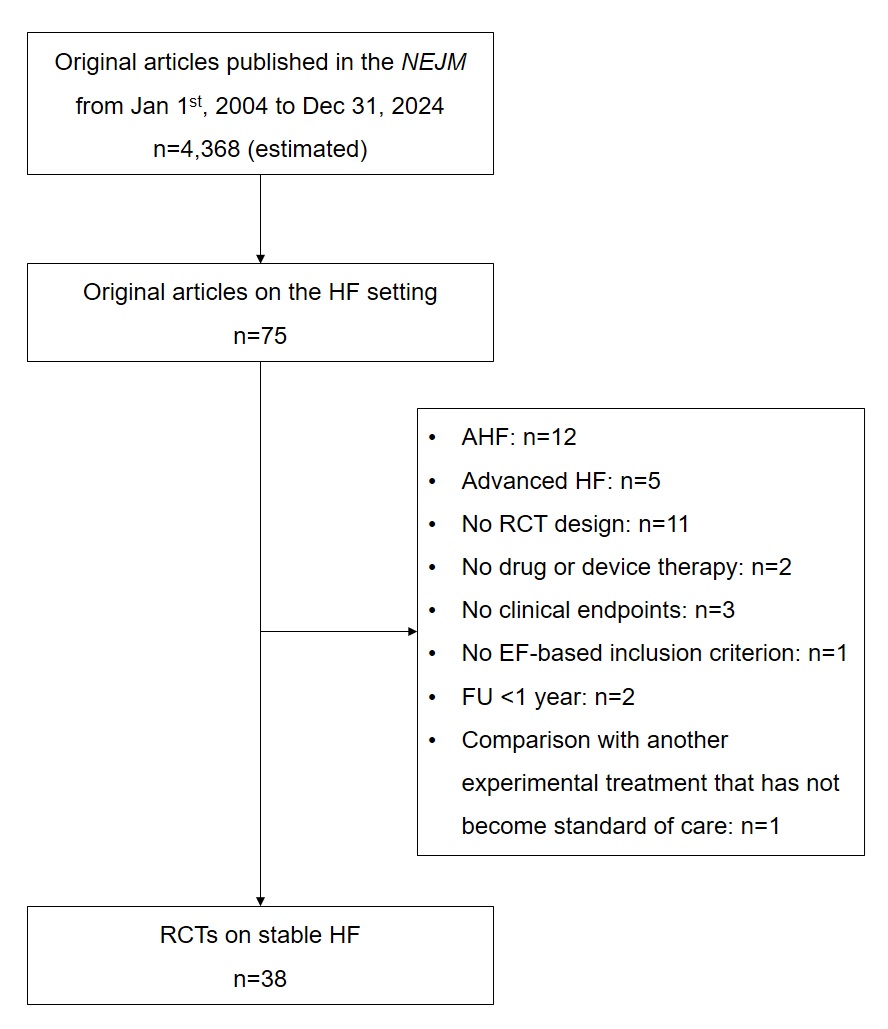


AHF, acute heart failure; EF, ejection fraction; HF, heart failure; RCT, randomized clinical trial.

**Supplementary Figure 2. Relationship between year of study start and annualized event rates in the control arm.**


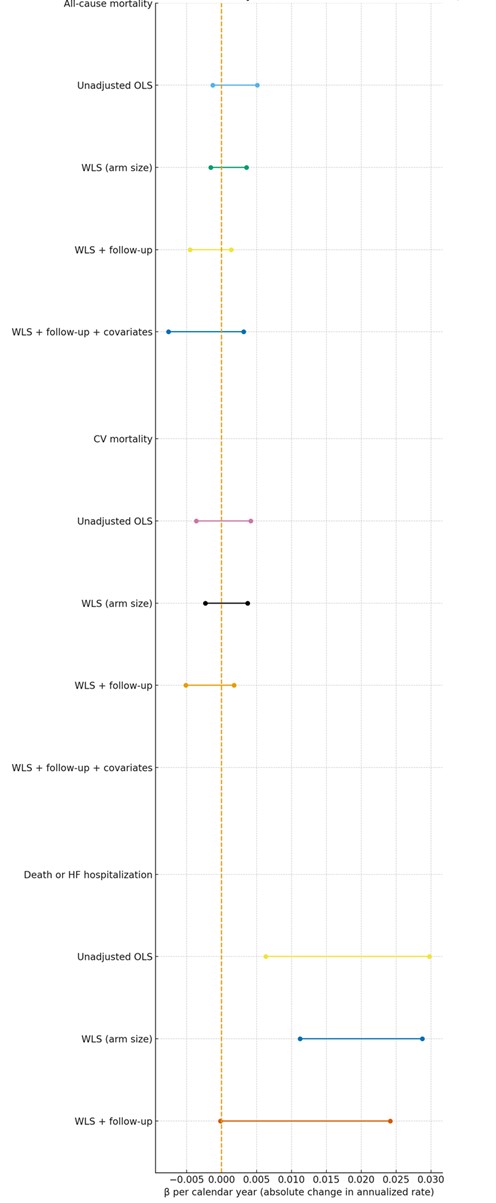


Points show beta (slope per calendar year) with 95% confidence interval (CI); horizontal lines are CIs and the dashed vertical line marks beta=0. Models: unadjusted ordinary least squares (OLS); weighted least squares (WLS; arm size) = weighted by arm sample size; WLS + follow-up (FU) = adds follow-up duration; WLS + FU + covariates = adds baseline clinical/treatment covariates (see Methods). Endpoints are all-cause mortality, cardiovascular (CV) mortality, and death or heart failure (HF) hospitalization.

**Supplementary Table 1. Randomized controlled trials on heart failure with reduced vs. mildly reduced or preserved ejection fraction (HFrEF vs. HFmrEF/HFpEF).**

|  | HFrEF  n=31 (80%) | HFmrEF/HFpEF  n=8 (20%) | p |
| --- | --- | --- | --- |
| Year of study start | 2008 (2002-2013) | 2018 (2008-2021) | 0.010 |
| Year of study publication | 2013 (2008-2018) | 2022 (2015-2024) | 0.015 |
| FU duration (years) | 2.3 (1.5-3.2) | 2.5 (2.1-3.2) | 0.550 |
| Patients treatment arm (n) | 659 (302-1,532) | 2,237 (704-3,002) | 0.057 |
| Patients control arm (n) | 602 (308-1,533) | 2,225 (706-2,996) | 0.040 |
| Age patients (years)* | 67 (63-69) | 72 (69-72) | 0.005 |
| Age controls (years)* | 67 (64-69) | 72 (69-72) | 0.008 |
| % female patients (%) | 23 (21-27) | 52 (45-57) | <0.001 |
| % female controls (%) | 24 (21-30) | 52 (45-55) | <0.001 |
| LVEF patients (years)* | 29 (25-31) | 57 (54-60) | <0.001 |
| LVEF controls (years)* | 28 (25-31) | 57 (54-59) | <0.001 |
| NT-proBNP patients (ng/L) | 1,759 (1,300-2,793) | 896 (374-1,038) | <0.001 |
| NT-proBNP controls (ng/L) | 1,700 (1,273-2,879) | 931 (365-1,025) | <0.001 |
| NYHA III-IV patients (%) | 46 (28-69) | 29 (21-33) | 0.062 |
| NYHA III-IV controls (%) | 46 (29-71) | 29 (19-36) | 0.073 |
| Beta-blockers patients (%) | 91 (82-93) | 79 (69-85) | 0.006 |
| Beta-blockers controls (%) | 91 (82-93) | 81 (73-85) | 0.010 |
| ACEi/ARB/ARNI patients (%) | 94 (89-97) | 81 (80-96) | 0.041 |
| ACEi/ARB/ARNI controls (%) | 93 (90-97) | 80 (78-83) | 0.002 |
| MRA patients (%) | 51 (41-60) | 37 (27-86) | 0.164 |
| MRA controls (%) | 52 (42-59) | 31 (4-38) | <0.001 |

Significant p values were reported in bold. Median and interquartile range were calculated from either mean or median values of continuous variables reported in each study. ACEi/ARB/ARNI, angiotensin converting enzyme inhibitor/angiotensin receptor blocker/angiotensin receptor/neprilysin inhibitor; FU, follow-up; LVEF, left ventricular ejection fraction; MRA, mineralocorticoid receptor antagonist; NT-proBNP, N-terminal pro-B-type natriuretic peptide; NYHA, New York Heart Association; SGLT2i, sodium-glucose cotransporter-2 inhibitor.

**Supplementary Table 2. Adoption of enrichment criteria over time.**

| Criterion | n | OR per decade | 95% CI (OR/decade) | p-value | PP change / year (pp/yr) | Prevalence (%) |
| --- | --- | --- | --- | --- | --- | --- |
| Age threshold ≥40y used | 31 | 0.42 | 0.16–1.07 | 0.068 | -0.48 | 6.4 |
| NYHA class III–IV required | 31 | 0.26 | 0.06–1.08 | 0.064 | -1.56 | 16.1 |
| Natriuretic peptide cut-off used | 31 | 14.09 | 1.93–102.75 | 0.009 | 4.49 | 35.5 |
| Recent HF event required | 31 | 5.6 | 0.83–37.62 | 0.076 | 2.66 | 22.6 |
| Pulmonary congestion/elevated filling pressures required | 31 | 0.08 | 0.02–0.31 | <0.001 | -0.52 | 3.2 |
|  | | | | | | |
|  | Beta per year (count) | 95% CI low | 95% CI high | p-value | R² | Change per decade (count) |
| N of criteria | 0.032 | -0.008 | 0.073 | 0.111 | 0.085 | 0.324 |

Analyses evaluate associations with calendar year of study start. Per-criterion trends are from logistic regression (odds ratio [OR] per decade); absolute percentage-point (pp) changes per year are from linear probability models. The count of criteria is modeled with ordinary least squares (OLS). OR per decade reflects the multiplicative change in odds of using a criterion per 10-year increase in calendar year. PP change/year is the absolute change in probability (percentage points) per calendar year. For the count model, beta is the absolute change in the number of criteria per year. CI, confidence interval; HF, heart failure; NYHA, New York Heart Association.

**Supplementary Table 3. Adoption of enrichment criteria (≤2008 vs >2008).**

| Criterion | ≤2008, n | ≤2008, % | >2008, n | >2008,  % | Risk diff  (>2008 vs. ≤2008, pp) | OR  (>2008 vs. ≤2008) | 95% CI (OR) low | 95% CI (OR) high | Fisher p |  |
| --- | --- | --- | --- | --- | --- | --- | --- | --- | --- | --- |
| Age threshold ≥40y used | 19 | 10.5 | 19 | 26.3 | 15.8 | 3.04 | 0.51 | 18.11 | 0.405 |  |
| NYHA class III–IV required | 19 | 26.3 | 19 | 0.0 | -26.3 | 0.07 | 0.00 | 1.32 | 0.046 |  |
| Natriuretic peptide cut-off used | 19 | 10.5 | 19 | 78.9 | 68.4 | 31.88 | 5.09 | 199.48 | <0.001 |  |
| Recent HF event required | 19 | 5.3 | 19 | 57.9 | 52.6 | 24.75 | 2.71 | 225.62 | 0.001 |  |
| Pulmonary congestion/elevated filling pressures required | 19 | 5.3 | 19 | 10.5 | 5.3 | 2.12 | 0.18 | 25.55 | 1.000 |  |
|  | | | | | | | | | | |
|  | Early n | Early mean±SD | Early median [IQR] | Late n | Late mean±SD | Late median [IQR] | Mean diff (Late–Early) | 95% CI (mean diff) | Welch t  p | Mann–Whitney p |
| Number of enrichment criteria (count) | 19 | 0.58±0.69 | 0.0 [0.0–1.0] | 19 | 1.42±0.77 | 1.0 [1.0–2.0] | 0.84 | 0.36 to 1.32 | 0.001 | 0.002 |

2008 was selected a priori as the dataset median year of study start, providing balanced groups and a data-driven era split that avoids *post-hoc* thresholding. CI, confidence interval; HF, heart failure; IQR, interquartile range; NYHA, New York Heart Association; OR, odds ratio; pp, percentage points.

**Supplementary Table 4. Add-one models.**

| Endpoint | Predictor | beta (predictor) | p (predictor) | ΔR² vs base | beta-year (base) | beta_year (add-one) | %Δbeta-year vs base |
| --- | --- | --- | --- | --- | --- | --- | --- |
| All-cause mortality | Age (years) | 0.0039 | 0.043 | 0.110 | -0.0016 | -0.0015 | -7.9% |
|  | Women (%) | -0.0010 | 0.381 | 0.022 | -0.0016 | -0.0018 | 11.0% |
|  | LVEF (%) | 0.0041 | 0.127 | 0.065 | -0.0016 | -0.0023 | 45.3% |
|  | log(NT-proBNP) | 0.0090 | 0.405 | 0.018 | -0.0016 | -0.0019 | 23.0% |
|  | NYHA III–IV (%) | 0.0006 | 0.087 | 0.078 | -0.0016 | -0.0009 | -46.1% |
|  | On beta-blocker (%) | -0.0009 | 0.606 | 0.005 | -0.0016 | -0.0005 | -68.1% |
|  | On ACEi/ARB/ARNI (%) | -0.0027 | 0.059 | 0.098 | -0.0016 | -0.0018 | 13.4% |
|  | On MRA (%) | 0.0001 | 0.878 | 0.059 | -0.0016 | -0.0027 | 74.0% |
|  | NP cut-off used (0/1) | -0.0310 | 0.160 | 0.055 | -0.0016 | 0.0005 | -134.0% |
|  | Recent HF required (0/1) | 0.0207 | 0.286 | 0.032 | -0.0016 | -0.0020 | 29.7% |
|  | NYHA III–IV required (0/1) | 0.0360 | 0.248 | 0.038 | -0.0016 | -0.0006 | -61.4% |
|  | Congestion/high filling P required (0/1) | 0.0122 | 0.804 | 0.002 | -0.0016 | -0.0015 | -4.3% |
|  | Age threshold ≥40y (0/1) | -0.0102 | 0.660 | 0.006 | -0.0016 | -0.0019 | 17.9% |
|  | Number of enrichment criteria | 0.0038 | 0.776 | 0.002 | -0.0016 | -0.0017 | 4.7% |
| CV mortality | Age (years) | 0.0016 | 0.545 | 0.018 | -0.0017 | -0.0011 | -31.3% |
|  | Women (%) | -0.0004 | 0.727 | 0.006 | -0.0017 | -0.0018 | 5.8% |
|  | LVEF (%) | 0.0037 | 0.206 | 0.075 | -0.0017 | -0.0019 | 16.0% |
|  | log(NT-proBNP) | 0.0081 | 0.439 | 0.204 | -0.0017 | -0.0006 | -67.0% |
|  | NYHA III–IV (%) | 0.0006 | 0.113 | 0.114 | -0.0017 | -0.0009 | -45.5% |
|  | On beta-blocker (%) | 0.0001 | 0.971 | 0.000 | -0.0017 | -0.0018 | 5.1% |
|  | On ACEi/ARB/ARNI (%) | -0.0016 | 0.409 | 0.033 | -0.0017 | -0.0015 | -7.4% |
|  | On MRA (%) | -0.0004 | 0.676 | 0.083 | -0.0017 | -0.0017 | 4.1% |
|  | NP cut-off used (0/1) | -0.0245 | 0.276 | 0.056 | -0.0017 | 0.0001 | -104.5% |
|  | Recent HF required (0/1) | 0.0176 | 0.397 | 0.035 | -0.0017 | -0.0019 | 15.0% |
|  | NYHA III–IV required (0/1) | 0.0642 | 0.073 | 0.143 | -0.0017 | -0.0003 | -81.3% |
|  | Congestion/high filling P required (0/1) | -0.0022 | 0.964 | 0.000 | -0.0017 | -0.0017 | 1.3% |
|  | Age threshold ≥40y (0/1) | -0.0358 | 0.138 | 0.101 | -0.0017 | -0.0033 | 98.4% |
|  | Number of enrichment criteria | -0.0041 | 0.776 | 0.004 | -0.0017 | -0.0016 | -2.2% |
| All-cause death or HF hospitalization | Age (years) | 0.0000 | 0.996 | 0.000 | 0.0120 | 0.0120 | 0.1% |
|  | Women (%) | 0.0003 | 0.934 | 0.000 | 0.0120 | 0.0122 | 2.1% |
|  | LVEF (%) | -0.0085 | 0.245 | 0.039 | 0.0120 | 0.0149 | 24.1% |
|  | NYHA III–IV (%) | 0.0002 | 0.854 | -0.012 | 0.0120 | 0.0119 | -0.8% |
|  | On beta-blocker (%) | -0.0062 | 0.422 | 0.009 | 0.0120 | 0.0175 | 46.3% |
|  | On ACEi/ARB/ARNI (%) | -0.0165 | 0.032 | 0.115 | 0.0120 | 0.0093 | -22.3% |
|  | On MRA (%) | -0.0038 | 0.277 | 0.082 | 0.0120 | 0.0102 | -14.7% |
|  | NP cut-off used (0/1) | -0.0686 | 0.333 | 0.028 | 0.0120 | 0.0151 | 25.8% |
|  | Recent HF required (0/1) | 0.0427 | 0.687 | 0.005 | 0.0120 | 0.0082 | -31.5% |
|  | NYHA III–IV required (0/1) | -0.0027 | 0.978 | 0.000 | 0.0120 | 0.0119 | -0.7% |
|  | Congestion/high filling P required (0/1) | 0.0000 | N/A | -0.000 | 0.0120 | 0.0120 | -0.0% |
|  | Age threshold ≥40y (0/1) | -0.0847 | 0.290 | 0.033 | 0.0120 | 0.0083 | -30.9% |
|  | Number of enrichment criteria | -0.0359 | 0.364 | 0.024 | 0.0120 | 0.0141 | 18.1% |

Weighted least squares (**WLS**) models of annualized **control-arm** event rates with weights equal to the number of control patients. The **base model** is endpoint ~ calendar year + follow-up duration; each **add-one model** adds a single predictor (listed under “Predictor”). **Beta (predictor)** is the change in the annualized event rate per unit increase in that predictor (continuous) or the difference vs reference (binary). **ΔR² vs base** is the increase in explained variance compared with the base model. **beta-year (base)** is the slope per calendar year in the base model; **beta_year (add-one)** is the slope after adding the predictor; **%Δbeta-year vs base** = 100×(beta_year(add-one) − beta-year(base)) / |beta-year(base)|, indicating attenuation (negative) or amplification (positive) of the year effect. Endpoints are **All-cause mortality**, **CV mortality**, and **All-cause death or HF hospitalization**. Follow-up refers to time to the primary endpoint. **ACEi/ARB/ARNI**, angiotensin-converting enzyme inhibitor/angiotensin receptor blocker/angiotensin-receptor neprilysin inhibitor; **CV**, cardiovascular; **HF**, heart failure; **LVEF**, left ventricular ejection fraction; **MRA**, mineralocorticoid receptor antagonist**; N/A, not available; NP**, natriuretic peptide; **NT-proBNP**, N-terminal pro-B-type natriuretic peptide; **NYHA**, New York Heart Association.

**Supplementary Table 5. Interaction models.**

| Endpoint | Predictor | beta (interaction) | p (interaction) | ΔR² vs base | beta-year (base) | beta-year at mean predictor |
| --- | --- | --- | --- | --- | --- | --- |
| All-cause mortality | Age (years) | 0.0003 | 0.420 | 0.126 | -0.0016 | -0.0015 |
|  | Women (%) | -0.0001 | 0.710 | 0.026 | -0.0016 | -0.0017 |
|  | LVEF (%) | -0.0002 | 0.687 | 0.069 | -0.0016 | -0.0023 |
|  | log(NT-proBNP) | 0.0060 | 0.003 | 0.448 | -0.0016 | -0.0001 |
|  | NYHA III–IV (%) | 0.0001 | 0.251 | 0.115 | -0.0016 | -0.0005 |
|  | On beta-blocker (%) | -0.0003 | 0.289 | 0.039 | -0.0016 | 0.0016 |
|  | On ACEi/ARB/ARNI (%) | 0.0000 | 0.898 | 0.099 | -0.0016 | -0.0018 |
|  | On MRA (%) | -0.0001 | 0.419 | 0.081 | -0.0016 | -0.0028 |
|  | NP cut-off used (0/1) | -0.0041 | 0.277 | 0.088 | -0.0016 | 0.0015 |
|  | Recent HF required (0/1) | -0.0052 | 0.237 | 0.072 | -0.0016 | -0.0026 |
|  | NYHA III–IV required (0/1) | 0.0091 | 0.207 | 0.082 | -0.0016 | 0.0004 |
|  | Congestion/high filling P required (0/1) | -0.0026 | 0.724 | 0.002 | -0.0016 | -0.0016 |
|  | Age threshold ≥40y (0/1) | -0.0123 | 0.390 | 0.027 | -0.0016 | -0.0027 |
|  | Number of enrichment criteria | -0.0003 | 0.899 | 0.003 | -0.0016 | -0.0016 |
| CV mortality | Age (years) | 0.0010 | 0.240 | 0.085 | -0.0017 | -0.0022 |
|  | Women (%) | -0.0006 | 0.258 | 0.070 | -0.0017 | -0.0021 |
|  | LVEF (%) | 0.0002 | 0.729 | 0.080 | -0.0017 | -0.0020 |
|  | log(NT-proBNP) | 0.0038 | 0.077 | 0.445 | -0.0017 | -0.0003 |
|  | NYHA III–IV (%) | 0.0001 | 0.499 | 0.134 | -0.0017 | -0.0005 |
|  | On beta-blocker (%) | -0.0003 | 0.564 | 0.017 | -0.0017 | -0.0001 |
|  | On ACEi/ARB/ARNI (%) | 0.0005 | 0.339 | 0.077 | -0.0017 | -0.0009 |
|  | On MRA (%) | -0.0001 | 0.420 | 0.120 | -0.0017 | -0.0016 |
|  | NP cut-off used (0/1) | -0.0031 | 0.494 | 0.079 | -0.0017 | 0.0010 |
|  | Recent HF required (0/1) | -0.0029 | 0.552 | 0.052 | -0.0017 | -0.0019 |
|  | NYHA III–IV required (0/1) | 0.0004 | 0.962 | 0.143 | -0.0017 | -0.0003 |
|  | Congestion/high filling P required (0/1) | -0.0006 | 0.928 | 0.000 | -0.0017 | -0.0017 |
|  | Age threshold ≥40y (0/1) | -0.0043 | 0.748 | 0.106 | -0.0017 | -0.0037 |
|  | Number of enrichment criteria | 0.0011 | 0.674 | 0.013 | -0.0017 | -0.0019 |
| All-cause death or HF hospitalization | Age (years) | 0.0046 | 0.058 | 0.101 | 0.0120 | 0.0083 |
|  | Women (%) | 0.0014 | 0.243 | 0.044 | 0.0120 | 0.0176 |
|  | LVEF (%) | -0.0012 | 0.481 | 0.053 | 0.0120 | 0.0153 |
|  | NYHA III–IV (%) | -0.0003 | 0.365 | 0.020 | 0.0120 | 0.0120 |
|  | On beta-blocker (%) | 0.0012 | 0.495 | 0.026 | 0.0120 | 0.0136 |
|  | On ACEi/ARB/ARNI (%) | -0.0025 | 0.010 | 0.220 | 0.0120 | 0.0082 |
|  | On MRA (%) | 0.0006 | 0.431 | 0.109 | 0.0120 | 0.0050 |
|  | NP cut-off used (0/1) | 0.0224 | 0.071 | 0.112 | 0.0120 | 0.0053 |
|  | Recent HF required (0/1) | 0.0660 | 0.002 | 0.202 | 0.0120 | 0.0118 |
|  | NYHA III–IV required (0/1) | -0.0233 | 0.379 | 0.026 | 0.0120 | 0.0097 |
|  | Congestion/high filling P required (0/1) | 0.0000 | nan | -0.000 | 0.0120 | 0.0120 |
|  | Age threshold ≥40y (0/1) | 0.0280 | 0.441 | 0.033 | 0.0120 | 0.0103 |
|  | Number of enrichment criteria | 0.0123 | 0.078 | 0.107 | 0.0120 | 0.0038 |

WLS models of annualized **control-arm** event rates testing **effect modification** of the calendar-year slope by each predictor: endpoint ~ year_c + follow-up + predictor_c + (year_c×predictor_c), where variables are mean-centered. **beta (interaction)** is the coefficient for **year×predictor**; **p (interaction)** tests whether the calendar-year slope differs by levels of the predictor. **ΔR² vs base** is the improvement in explained variance over the base model (endpoint ~ year + follow-up). **beta-year (base)** is the calendar-year slope in the base model; **beta-year at mean predictor** is the calendar-year slope evaluated at the sample-mean value of the predictor. Endpoints and follow-up definitions are as above. **ACEi/ARB/ARNI**, angiotensin-converting enzyme inhibitor/angiotensin receptor blocker/angiotensin-receptor neprilysin inhibitor; **CV**, cardiovascular; **HF**, heart failure; **LVEF**, left ventricular ejection fraction; **MRA**, mineralocorticoid receptor antagonist**; N/A, not available; NP**, natriuretic peptide; **NT-proBNP**, N-terminal pro-B-type natriuretic peptide; **NYHA**, New York Heart Association.
